# A paramyxovirus-like model for Ebola virus bipartite promoters

**DOI:** 10.1371/journal.ppat.1008972

**Published:** 2020-11-05

**Authors:** Irina Gutsche, Philippe le Mercier, Daniel Kolakofsky

**Affiliations:** 1 Institut de Biologie Structurale, Univ Grenoble Alpes, CEA, CNRS, IBS, Grenoble, France; 2 Swiss-Prot Group, Swiss Institute of Bioinformatics, Centre Médicale Universitaire, Geneva, Switzerland; 3 Department of Microbiology and Molecular Medicine, University of Geneva Medical School, Geneva, Switzerland; University of Pittsburgh, UNITED STATES

## Abstract

Paramyxo- and filovirus nucleocapsids (NCs) have bipartite promoters at their 3′ ends to initiate RNA synthesis. The 2 elements, promoter element 1 (PE1) and promoter element 2 (PE2), are separated by a spacer region that must be exactly a multiple of 6 nucleotides (nt) long. Paramyxovirus NCs have 13 nucleoprotein (NP) subunits/turn, such that PE1 and PE2 are juxtaposed on the same face of the NC helix, for concerted recognition by the viral polymerase. Ebola virus (EBOV) NCs, in contrast, have 25 to 28 subunits/turn, meaning that PE1 and PE2 cannot be juxtaposed. However, there is evidence that the number of subunits/turn at the 3′ end of the EBOV NC is variable. We propose a paramyxovirus-like model for EBOV explaining why there are 8 contiguous copies of the PE2 repeat when 3 are sufficient, why expanding this run to 13 further improves minigenome performance, and why there is a limit to the number of hexa-nt that can be inserted in the spacer region.

Non-segmented negative-strand RNA virus (nsNSV) genomes are 12 to 20 kB in length and contain 5 to 10 tandemly arranged genes. There are 4 main families: Rhabdoviridae (vesicular stomatitis virus (VSV) and rabies), Paramyxoviridae (Sendai virus (SeV) and measles), Pneumoviridae (respiratory syncytial virus (RSV)), and Filoviridae (Ebola virus (EBOV) and Marburg virus (MARV)). nsNSV genome RNAs are enclosed within a non-covalent polymer of nucleoprotein (NP) to form a helical nucleocapsid (NC), the substrate for all viral RNA synthesis. The NP subunits of paramyxo- and filovirus NCs bind precisely 6 nucleotides (nt) each, with 3 contiguous bases pointing toward the protein core and 3 pointing away, whereas those of pneumo- and rhabdoviruses bind 7 and 9 nt/subunit, respectively. Paramyxo- and filoviruses also differ from the other 2 families in that their mRNAs can be co-transcriptionally edited to express alternate open reading frames (ORFs). EBOV edits its glycoprotein mRNA to make the essential full-length glycoprotein, and MARV (a member of another filovirus genus), which does not need to edit its glycoprotein mRNA, may edit its NP and polymerase (L) mRNAs [1]. Furthermore, paramyxo- and filovirus genomes have bipartite promoters at their 3′ ends to initiate RNA synthesis. There is both a 3′ promoter element (PE1) within the short leader region, and an internal element (promoter element 2 (PE2)) within the 5′ UTR of the first gene. PE1 and PE2 are separated by a spacer region that includes the first mRNA start site near position 56 from the genome 3′ end. Initiation of RNA synthesis from this end not only requires both elements but also that they be separated by a spacer region that is a multiple of 6 nt long. The insertion or deletion of even a single nt here (or a stretch of RNA that is not a multiple of 6 nt long) will inactivate these promoters [2–5].

Paramyxovirus genome replication is governed by the “rule of six” [6], i.e., all paramyxovirus genomes found in nature are a multiple of 6 nt long, and only minigenomes of hexamer length replicate well in cell culture. This rule imposes a hexamer phase on the entire genome, which is composed of a series of hexa-nt bound to each NP subunit. Paramyxovirus PE2 simply consists of 3 contiguous hexamers where only 1 or 2 of these nt are at all important, e.g., 3′-CN_5_ in NPs number 14–16 (NP#14–16, counting from the genome 3′ end) for SeV, and 3′-N_4_GC in NP#13–15 for parainfluenza virus type 5 (PIV5) [2,7]. Because paramyxovirus NCs contain approximately 13 NP subunits/turn [8–12], these tripartite PE2s are juxtaposed on the same axial face of the NC helix at the 3′ end of the genome, presumably for concerted recognition by the viral polymerase [13,14]. The manner in which this bipartite promoter operates was recently examined for PIV2, a close relative of PIV5. Ten residues of their NP RNA-binding groove make contact with the RNA, 9 with the ribose-PO_4_ backbone, and 1, gln202, with an nt base modeled as uracil [15]. Minigenomes containing wild-type (wt) NP-Q202 require both PE1 and PE2, as well as hexamer-length genomes for activity (i.e., wt hexamer phasing). However, when Q202 is mutated to 1 of several other residues, PE2 is no longer required at all, and non-hexamer-length minigenomes remain active [16]. PE1 was proposed to contain a negative element, namely Q202 making base-specific contact with the genome 3′ uridine, where all RNA synthesis likely begins. This interaction presumably prevents promoter activity unless polymerase can also make contact with the correctly phased PE2 tripartite repeat [17]. In this manner, paramyxovirus bipartite promoters ensure the hexamer phase of the entire genome, including that of the *cis*-acting mRNA editing signal where hexamer phase participates in regulating this process [18].

Paramyxo- and filoviruses share many properties, but there are also important differences. EBOV actually acts like a pair of viruses, whose overall genome lengths differ by a single nt because their mRNA editing site can contain a run of either 7 or 8 uridines (EBOV/7U and EBOV/8U); both genomes are present during infection, regardless of which EBOV has initiated the infection [19]. Moreover, EBOV and MARV minigenomes are indifferent to their overall length, even though their bipartite promoters must obey the same rules of PE2 hexamer phasing as those of paramyxoviruses [5,20,21]. Remarkably, EBOV Zaire (or ZEBOV) contain 8 contiguous copies of its PE2 hexamer repeat (3′-NNURRN for EBOV/8U that is of hexamer length and 3′ NNNURR for EBOV/7U) [22]. A minimum of 3 are still active, and the promoter gains strength when more hexamer repeats are present [5]. MARV in contrast contains the apparent minimum of 3 contiguous hexamer repeats [23]. Notably, the run of 8 hexamer repeats is not conserved in the 5 other species of the EBOV genus; EBOV Bundibugyo virus (BDBV) and Tai Forest virus (TAFV) have 6, Sudan virus (SUDV) and Reston virus (RESTV) have 5, and Bombali virus (BOMV) has 4 (Fig 1). When ZEBOV’s run of 8 hexamer repeats is extended into the leader region (via a single base change) to 13 contiguous repeats, this further improves minigenome reporter gene expression. Most remarkably, this spacer region can be expanded to accommodate as many as 8 or 9 additional hexa-nt, as long as the hexamer phase of the PE2 repeats remains the same [20], and BOMV, in fact, appears to have included 4 additional hexa-nt between the almost perfectly conserved leader plus gene start site and the hexamer repeats. This is in strong contrast to paramyxovirus bipartite promoters where displacement of the 3 PE2 hexamers by deletion of 6 nt in the spacer region between PE1 and PE2 abolished SeV and PIV5 minigenome replication [2,4]. For SeV, 6 nt insertions in this region of the promoter were tolerated, but insertion of 12 nt largely ablated promoter activity [24]. Thus, paramyxovirus bipartite promoters have a more limited tolerance for alteration of the spacing of the 2 promoter elements. The superabundance of EBOV PE2 hexamer repeats, and the tolerance of the hexa-nt length of the spacer region suggests that the NC structure recognized by EBOV polymerase for initiation of RNA synthesis is unlikely to be unique or fixed, as suggested for paramyxoviruses.

**Fig 1:**
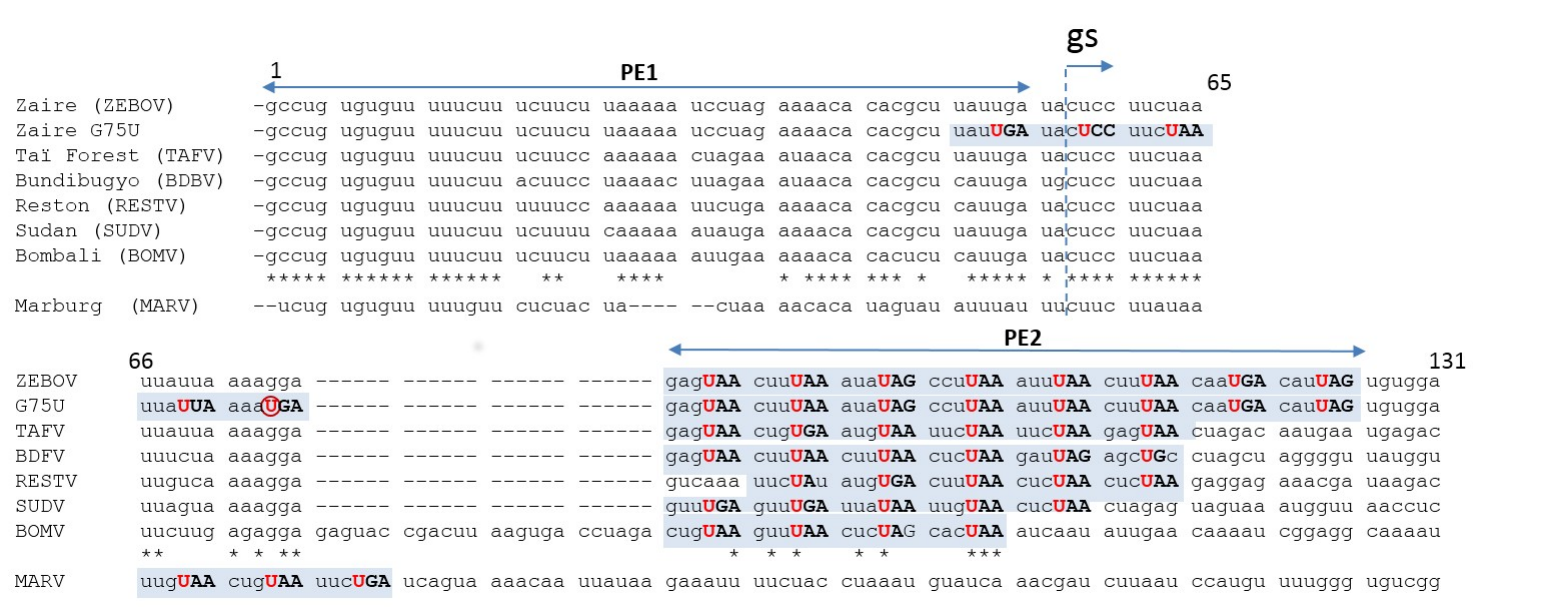
Filovirus bipartite promoters. This alignment of the EBOV/U7 genome 3′ ends is based on the premise that genomes are assembled directly from their 5′ ends, 6 nt/NP protomer, generating genome hexamer phase where only nt in hexamer positions 3, 4, and 5 point away from the protein core and can readily interact with the polymerase (underlined below). At least for ZEBOV, sequences upstream of the editing site will have different hexamer phases, e.g., 3′ NNN**U**RR for EBOV/U7 and 3′ NN**U**RRN for EBOV/U8 for the PE2 repeats. The conserved 3′ URR of contiguous PE2 repeats (shaded in blue) is capitalized. The conserved U of the repeat is highlighted in red and that of G75U is circled, and asterisks below the EBOV sequences indicate their overall sequence conservation. MARV, aligned below, does not appear to have any spacer region between gs (indicated by bent arrow) and the 3 PE2 repeats. Only the ZEBOV and MARV PE2 repeats have been experimentally verified [23]. BDBV, Bundibugyo virus; BOMV, Bombali virus; EBOV, Ebola virus; gs, gene start; MARV, Marburg virus; NP, nucleoprotein; nt, nucleotides; PE1, promoter element 1; PE2, promoter element 2; RESTV, Reston virus; SUDV, Sudan virus; TAFV, Tai Forest virus; ZEBOV, EBOV Zaire.

If paramyxo- and filoviruses evolved from a common ancestor and have adopted bipartite promoters to assure genome hexamer phasing (in large part to regulate mRNA editing), we would expect their bipartite promoters to operate similarly to the extent that their biology permits. The EBOV model we recently presented based on a 13 NP/turn helix, for which there is no evidence of its existence, is more than questionable and does not explain the filovirus data [20,22], while remaining valid for paramyxoviruses. Recent cryo-electron tomography structures of EBOV NCs within intact viruses and recombinant NC-like assemblies allow estimating their approximate helical symmetries from subtomogram averaging and show a variable number of subunits per turn ranging between roughly 25 and 28 [25]. This assembly, however flexible, does not lend itself to a paramyxovirus-like model (polymerase’s simultaneous interaction of PE1 and PE2), as the 8 PE2 hexamer repeats are in NP#14–21 from the 3′ end. A paramyxovirus-like model of interaction would require abandoning the notion of a uniform EBOV NC structure that includes the genome 3′ end. If PE1 and PE2 need to be juxtaposed on the first 2 turns of the helix so that the polymerase can interact with both (and judge the intervening sequence length to within a single nt), this requires much more helix plasticity for the 3′ end of the EBOV NC, and here, another nsNSV may be instructive.

Rhabdo- and filoviruses form rod-like virions whose length is determined by that of their NC, whereas paramyxo- and pneumovirus NC are contained within spherical envelopes. The 3′ end of the VSV NC, at the pointed end of the bullet-like virion, is itself also pointed or conical. Its tip (the 3′-most turn) likely consists of around 10 subunits/turn and increases to the uniform 33 subunits/turn of the NC trunk after 5 to 6 turns [26]. Remarkably, the formation of this conical tip of the VSV NC turns out to be an inherent property of its NP [27]. Electron microscopy images of the EBOV and MARV particles show that the tip of the virus and the visible NC filament within is actually also conical [28,29], but until now, all structural studies were focused on the straightest and most ordered helical portion of the EBOV NC [25,28,30]. While the structure at the tip of the NC has not as yet been resolved, the variation in the number of subunits per turn of VSV NC also occurs at the 3′ end of EBOV NCs, at least inside the virus particle. VSV NCs within virions are of course fixed structures, whereas EBOV NCs within the cell cytoplasm, perhaps with polymerase in place to initiate RNA synthesis, need not be fixed. In such a scheme for EBOV, the diameter of the first turn of the helix may be variable and dynamic. The G75U mutant of Bach and colleagues [20] offers some insights into the structural plasticity of the EBOV tip enabling alignment of PE1 and PE2 for promoter activation. Due to this single base change in this mutant, the contiguous stretch of 8 3′ NNURRN repeats (NP#14–21) is extended to 13 (NP#9–21) (Fig 1). If the lower limit of the first turn of the helix were 10 subunits/turn similarly to VSV NC, the tripartite repeats of NP#11–13 would align with NP#1–3 on the first 2 turns of the helix for activity (Fig 2A), which may explain why the G75U mutant outperforms the wt minigenome [20]. When there are 13 subunits/first turn, the first of the wt tripartite repeats (NP#14–16) would align (Fig 2B). When there are 14 subunits/first turn, the second of the wt tripartite repeats (NP#15–17) would align, etc., and when there are 18 subunits/first turn, the last of the wt tripartite repeats (NP#19–21) would align (Fig 2C). Notably, in this last case of 18 subunits/first turn, the insertion of additional hexa-nt to the spacer region would simply displace NP#19–21 downstream, to be replaced by the more 3′ tripartite repeats, until NP#14–16 are aligned with the 3′ end (after the addition of 5 hexa-nt to the spacer region (Fig 2D)). The addition of more than 5 hexa-nt to the spacer in this case leads to a loss of promoter activity because PE2 can no longer align with PE1, and this places a limit on the length of RNA that can be added to the spacer [20]. The variable length of the first turn allows all 8 of the wt repeats and 3 of the additional repeats of the G75U mutant to participate in the alignment of PE1 and PE2. If the first 2 turns of the helix are dynamic, with the PE1-contacting region of the L polymerase fixed near the 3′ end so that it can negatively control the promoter, the PE2-contacting region of L can scan for an NC region with the correctly phased PE2 tripartite repeat. Only upon this concerted interaction can the negative effect on PE1 be overcome and RNA synthesis initiate. In this way, paramyxo- and filovirus bipartite promoters would operate similarly, even though EBOV genomes need not, nor cannot, given their biology, always be of hexamer length (e.g., EBOV/7U is 6n+5 nt long, (Fig 1)). As for MARV that contains the apparent minimum of 3 contiguous PE2 hexamer repeats, it may not require this degree of promoter plasticity [21,23].

**Fig 2:**
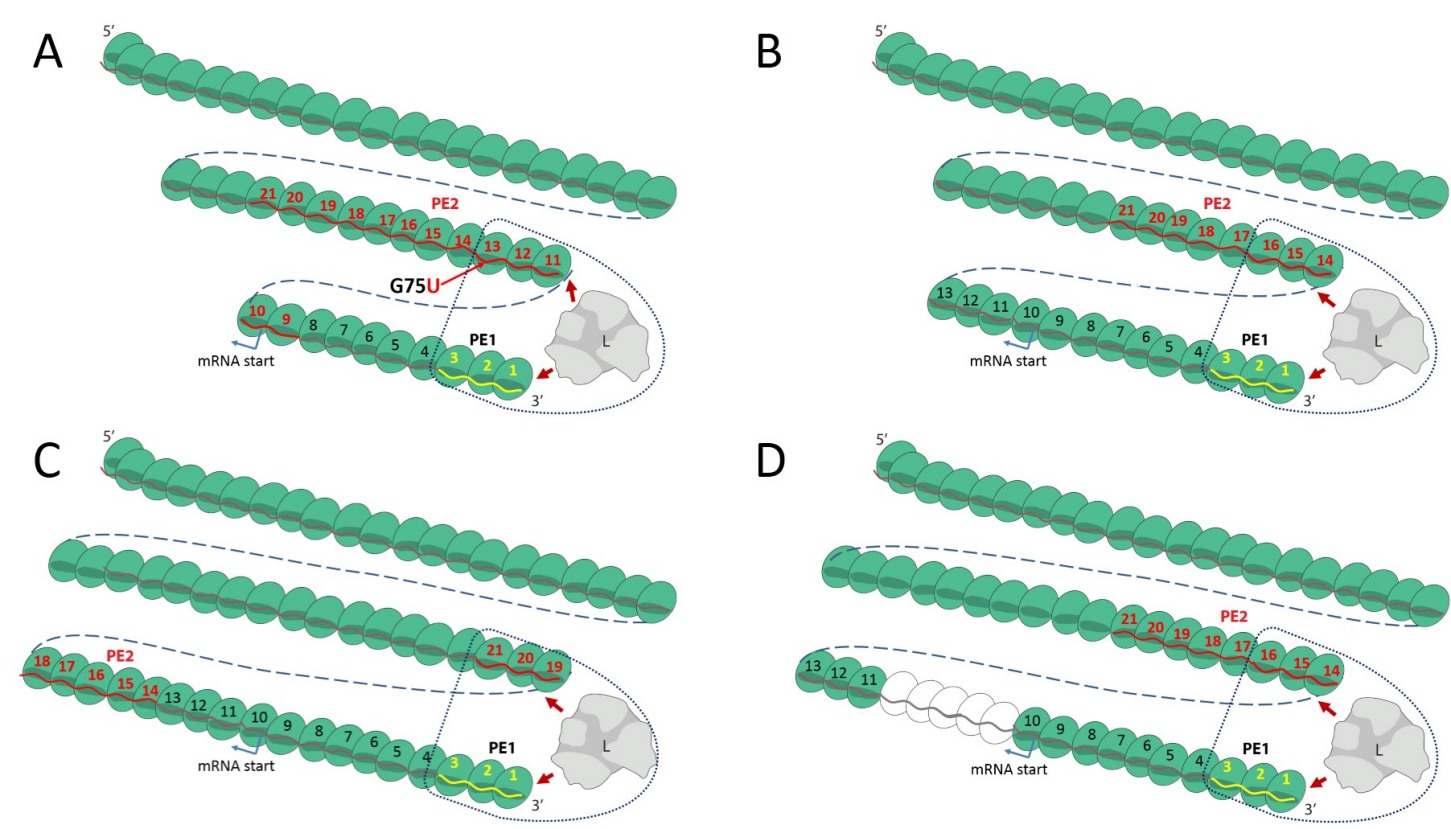
A paramyxovirus-like model for EBOV bipartite promoters, based on a variable and dynamic 3′ end of the NC helix. The G75U and wt NC helices are schematically shown as 2-dimensional chains of NP subunits (green spheres), numbered from their 3′ ends, whose first turn is either 10 (panel A), 13 (panel B), or 18 subunits long (panels C and D). The open spheres in panel D represent hexa-nt insertions in the wt minigenome. Their genome RNAs are shown as a wavy line within their NP RNA-binding grooves (darker green). PE1, modeled as the first 3 subunits, is highlighted in yellow. Subunits containing contiguous PE2 hexamer repeats (3′ NN**U**RRN for EBOV/8U and 3′ NNN**U**RR for EBOV/7U) are highlighted in red. The polymerase (L) is shown in gray, interacting with PE1 and the minimum PE2 tripartite repeat needed for the initiation of RNA synthesis. For further detail, see text. EBOV, Ebola virus; NC, nucleocapsid; NP, nucleoprotein; nt, nucleotides; PE1, promoter element 1; PE2, promoter element 2; wt, wild-type.
